# Insulin-Like Growth Factors, Binding Proteins and Their Role in Pregnancy in Patients With Diabetes

**DOI:** 10.1155/jdr/3330482

**Published:** 2025-11-24

**Authors:** Aleksandra Gladych-Macioszek, Katarzyna Ozegowska, Sandra Radzicka-Mularczyk, Rafał Sibiak, Gernot Desoye, Ewa Wender-Ozegowska

**Affiliations:** ^1^Department of Reproduction, Chair of Fetomaternal Medicine, Poznan University of Medical Sciences, Poznan, Poland; ^2^Doctoral School, Poznan University of Medical Sciences, Poznan, Poland; ^3^University Unit of Treatment and Diagnostics for Infertility, Poznan University of Medical Sciences, Poznan, Poland; ^4^Department of Obstetrics and Gynecology, Medical University of Graz, Graz, Austria

**Keywords:** adverse pregnancy outcomes, diabetes, insulin-like growth factor, insulin-like growth factor–binding protein

## Abstract

The insulin-like growth factor (IGF) axis, an evolutionarily conserved system, is critical in regulating cell growth, proliferation, and survival, affecting nearly all organ systems. This axis comprises two growth factors, IGF-I and IGF-II, and six insulin-like growth factor–binding proteins (IGFBPs), modulating IGF activity. Due to their structural similarity to insulin, IGFs can interact with insulin receptors, facilitating glucose uptake in adipose and muscle tissues, suppressing hepatic glucose production, and modulating blood glucose levels. Pregnancy induces unique metabolic challenges, with the IGF axis adapting to support maternal metabolic changes and fetal growth. In pregnancies complicated by prediabetes or diabetes, disruptions in the IGF axis, including altered levels of IGFs and IGFBPs, have been linked to adverse outcomes such as macrosomia and intrauterine growth restriction. This review discusses the role of the IGF system in pregnancies with diabetes, focusing on how dysregulation of IGFs and IGFBPs contributes to pregnancy complications. We emphasize the dual role of the IGF axis in metabolism and growth and evaluate its potential as a therapeutic target in managing high-risk pregnancies.

## 1. Introduction

Pregnancy is a complex process that involves numerous adjustments to provide a consistent supply of essential nutrients to support the fetus's growth and development. The woman's body undergoes widespread changes during pregnancy, impacting nearly every system. Among others, these changes include increased insulin resistance, altered carbohydrate metabolism, and increased insulin production. It is a complex process involving continuous adaptations and metabolic shifts [1].

In the transition period from the first to the second half of pregnancy, maternal tissues gradually become insulin resistant, that is, less responsive to insulin, accompanied by a rise in fasting insulin concentrations, to ensure sufficient nutrients are directed to the developing fetus [2, 3]. The actions of insulin-like growth factors (IGFs) may reflect metabolic disturbances and can act as a compensatory mechanism [4]. Due to their structural similarity to insulin, IGFs can bind to insulin receptors (IRs) with different affinity [5, 6]. The mother's insulin resistance level and associated hyperglycemia are linked to the amount of glucose transferred from her to the fetus [7, 8]. During pregnancy, changes in carbohydrate metabolism are supported by increased insulin production and heightened resistance to its effects. This involves reduced sensitivity to the usual hormonal regulation and a diminished capacity for maximum insulin response [9].

Maternal hyperglycemia results in elevated fetal blood glucose and fetal hyperinsulinemia, leading to excessive fetal growth and, particularly, fat accumulation, often represented by fetal macrosomia—one of the most frequent and significant complications of maternal diabetes and obesity during pregnancy. Macrosomia is associated with a higher likelihood of in utero death, instrumental delivery, shoulder dystocia, and later-life obesity, birth canal trauma, and an increased rate of Caesarean sections among hyperglycemic patients. Fetal growth is a complex process influenced by genetics, maternal health conditions, the intrauterine environment, and fetal hormones. Differences in maternal glycemia, as assessed by HbA1c, mainly in the second trimester, may explain some differences in a newborn's birth weight [10]. Additionally, the impact of growth factors, both maternal and fetal, may be of importance.

Most recent data show that not only short-term consequences are significant, but in the long term, adult diseases might also have their origin in fetal life. In particular, the late complications of hyperglycemia in pregnancy relate not only to the increased risk of diabetes itself [11] but also to the risk of cardiovascular disease [6], kidney [7] and liver disease [8, 9], neurodevelopmental disorders [11], or infertility problems [12].

The placenta forms the crucial connection between mother and fetus, facilitating oxygen and nutrient transfer and supporting fetal growth during pregnancy [13]. Following a concentration gradient between mother and fetus, transplacental glucose transport occurs through facilitated diffusion, mediated by members of the glucose transporter (GLUT) family [14]. IGFs and related proteins are also present in the placenta and are supposed to be essential in driving maternal adaptations and fetal growth and development [15, 16].

Insulin-like growth factors and their type 1 receptor (IGF1R) are among the earliest expressed genes in the inner cell mass of the developing embryo, underscoring their critical role in early embryonic development [17, 18]. Early research indicated that IGF-I, one of two growth factors of the IGF family, functions as a mitogen and differentiation agent in preimplantation embryos [19] and in cultured fetal cells [20–22]. Cultured fetal cells and tissue explants secrete IGFs in vitro [23].

A distinctive aspect of the IGF axis in human reproduction is the parental imprinting of several IGF-related genes, where gene expression is limited to maternal or paternal alleles. This epigenetic mechanism is vital for early embryonic development and function. For instance, IGF-II, primarily expressed from the paternal allele, is crucial for promoting placental trophoblast proliferation and enhancing nutrient transport capacity, both essential for fetal growth [24]. On the other hand, the maternally expressed IGF2 receptor (IGF2R) moderates IGF-II activity by acting as a reservoir to capture excess ligands, thereby maintaining a balance in its growth-promoting effects [25].

IGF-I has both mitogenic and anabolic actions [26]. The biological actions of the IGFs are regulated at the level of bioavailability through association with a family of specific high-affinity insulin-like growth factor–binding proteins (IGFBPs). The ligand binding affinity of the IGFBPs can be modulated by phosphorylation and proteolysis. IGF-I concentrations in maternal serum decrease between 6 and 12 weeks in diabetic pregnancies compared to nondiabetic controls [27]. Cord serum concentrations of IGF-I are increased in newborns of insulin-treated mothers (Type 1 diabetes [T1D] and Type 2 diabetes [T2D]) compared to nondiabetic controls [28]. Moreover, IGF-II cord serum levels are increased in pregnancies with diabetes but without correlation with infant birth weight [29].

This review is aimed at providing an overview of the roles of IGFs and IGFBPs in pregnancy, focusing on patients with both gestational and pregestational diabetes. We will cover how these factors influence maternal and fetal outcomes and examine their potential as therapeutic targets or biomarkers for managing diabetic pregnancies. Understanding the roles of IGFs and IGFBPs in this context is crucial for improving maternal and neonatal care in diabetic pregnancies.

## 2. Materials and Methods

A comprehensive, nonsystematic, narrative review of the international literature examined the role of IGFs and their binding proteins in pregnancy complicated with pregestational diabetes mellitus (PGDM) and gestational diabetes mellitus (GDM). The researchers reviewed publications from the PubMed database, targeting studies from the first records in 1983 until 2024. The review focused on English-language publications. Keywords used in the search strategy were “Insulin-Like Growth Factor binding protein,” “Insulin-Like Growth Factor,” “diabetes,” “pregnancy,” “placenta,” “gestational diabetes,” “pregestational diabetes,” and “adverse pregnancy outcomes.”

## 3. IGFs and Their Binding Proteins

**Figure 1:**
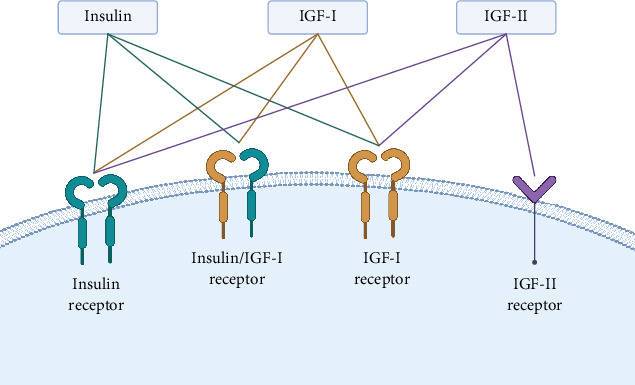
IGF-related ligands and their receptors. Abbreviations: IGF-I—insulin-like growth factor I, IGF-II—insulin-like growth factor II. Created in BioRender. Sibiak, R. (2025), https://BioRender.com/o67u620.

**Figure 2:**
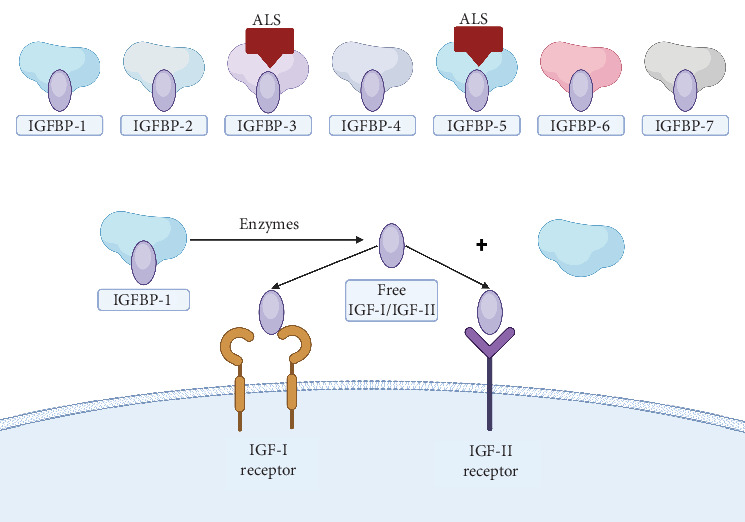
IGF binding proteins and their complexes. Circulating IGFs are shielded from degradation by forming complexes with IGFBPs. They can be found in the bloodstream as binary complexes with individual IGFBPs. Additionally, ternary complexes are formed when binary complexes involving IGFBP-3 or IGFBP-5 bind to the acid-labile subunit (ALS). Free IGF-I/IGF-II interacts with cell surface receptors. Abbreviations: IGFBP—insulin-like growth factor binding protein; ALS—acid-labile subunit. Created in BioRender. Sibiak, R. (2025), https://BioRender.com/e38c140.

The IGF system is a biologically conserved pathway consisting of two IGF ligands, their receptors (IGF-IR, IGF2R, IR, and the hybrid IR/IGF-IR), and six IGFBPs [30] (Figure 1). IGF-I and IGF-II, the IGFs, are two small, closely related single-chain polypeptides [31]. First identified in 1957, they were initially named sulfation factors and later renamed somatomedins and IGFs. There are also six high-affinity binding proteins (IGFBP 1–6) and their enzymes [32] (Figure 2). Since their initial characterization, IGFs have been shown to share a high degree of homology with insulin [33].

### 3.1. IGF-I and Its Receptors

The IGF-I gene is located on the long arm of Chromosome 12. It has a complex structure with multiple promoters and contains six exons, four of which are subjected to alternative splicing. IGF expression is finely regulated at transcription, RNA processing, and translation levels, and the IGF-I precursor and IGF-II precursor undergo proteolytic processing at both ends [34]. IGF-I is a 70-amino acid peptide produced by most tissues throughout the body. IGF-I functions as a circulating hormone and a local tissue growth factor. Circulating IGF-I is derived from the liver, although other tissues may contribute [35]. The main factor that regulates hepatic IGF-I biosynthesis is growth hormone (GH). In nonhepatic tissues, IGF-I gene expression can be controlled by factors besides GH, like parathyroid hormone, prostaglandin E2 [36], and estradiol [37]. More than 75% of IGF-I is confined to the vascular compartment as a ternary complex with the acid-labile subunit (ALS) and IGFBP-3, the most abundant circulating IGFBP [38]. When this complex breaks apart, IGFs bind with other IGFBPs to form smaller binary complexes, facilitating IGF transport across the endothelium to target tissues. IGF-I binds with high affinity to IGF-IR, a family of tyrosine kinase receptors. IGF-IR is expressed in every human tissue and cell type [31]. Upon ligand binding, the intracellular tyrosine kinase domain undergoes autophosphorylation at specific tyrosine residues, creating docking sites for signaling proteins like the insulin receptor substrate (IRS) family. These proteins then recruit additional substrates, activating various signaling pathways that promote cell proliferation and survival [39]. IGF-I can also bind to IRs. IGF-IR and IR are highly similar, with 60% amino acid homology. Both comprise *α*- and *β*-subunits, with the *α*-subunit located entirely extracellularly and the transmembrane *β*-subunit having large intracellular domains [40]. Due to their homology, IGF-IR and IR can merge and form hybrid receptors [41]. Hybrids comprising an IR *α*-subunit and an IGF-IR *β*-subunit can bind IGF-I, IGF-II, and insulin. In contrast, hybrids composed of an IR *β*-subunit and an IGF-IR *α*-subunit respond only to IGF-I but not IGF-II or insulin [42]. While insulin binds to IGF-IR at supra high, nonphysiological concentrations in vitro, there is limited evidence to support significant binding under physiological conditions in vivo [42, 43].

### 3.2. IGF-II and Its Receptors

The IGF-II gene is part of a gene cluster on the short arm of Chromosome 11. It is structurally homologous to proinsulin and IGF-I. IGF-II is a 67-amino acid, single-chain polypeptide in the circulation. IGF-II expression in cultured cells is regulated by various factors depending on cell type. In ovarian cells, follicle-stimulating hormone (FSH), chorionic gonadotropin, and cyclic AMP (cAMP) modulate IGF-II expression; adrenocorticotropic hormone (ACTH) and cAMP in fetal adrenal cells; glucocorticoids and thyroglobulin in hepatic cells; and glucose in a pancreatic beta-cell line. IGF-II levels rise in response to glucose in fetal hepatocytes and play a key autocrine/paracrine role in promoting skeletal muscle myoblast differentiation [31, 44]. IGF-II binds to IGF2R, widely distributed across human tissues, with a shortened, soluble form of the receptor circulating in the bloodstream [45]. IGF-II also binds to the IGF-IR, though with six times less affinity than IGF-I, while insulin binds with an affinity 100 times lower to the IGF-IR [31, 32].

In addition to binding IGF-II, the primary functions of IGF2R include sorting newly synthesized lysosomal enzymes and facilitating endocytosis of extracellular lysosomal enzymes [46]. To carry out these distinct functions, the extracellular region of the receptor contains binding sites for both IGF-II and phosphomannosyl residues [47]. In mammals, the activity of IGF-II, but not of IGF-I, is additionally regulated by IGF2R, which sequesters IGF-II for internalization and degradation. IGF2R functions as a growth inhibitor, and its loss leads to enhanced growth [48] (Figure 1).

### 3.3. IGFBPs

IGFBPs are IGF bioactivity's flexible endocrine and autocrine/paracrine regulators. IGFBP family members include IGFBP-1, IGFBP-2, IGFBP-3, IGFBP-4, IGFBP-5, and IGFBP-6. IGFBPs have higher affinities for IGFs than IGF-IR. Therefore, IGFBPs act not only as carriers of IGFs, thereby prolonging their half-life, but also function as modulators of IGF availability and activity [49, 50]. About 90% of circulating IGFs are bound to IGFBPs, mostly in 50-kDa binary complexes. IGFBP-3 or IGFBP-5 is part of ternary complexes with IGF and the ALS, making up around 75% of circulating IGFBP3 or IGFBP5 (Figure 2). Formation and dynamics of these complexes are particularly relevant in pregnancy due to altered IGFBP proteolysis and hormonal regulation [38].

IGFBP genes have a conserved genomic structure, each with four exons except IGFBP-3, which also has a 3⁣′ noncoding exon. Human IGFBPs 1–6 consist of 216–289 amino acids, organized in three domains: N-terminal, linker, and C-terminal, each of roughly equal length. IGFBPs could act in an IGF-dependent or IGF-independent manner. They physically block the interaction between IGFs and the IGF-IR, acting as competitive solid inhibitors by binding IGFs with approximately 10 times greater affinity than the IGF-IR. They inhibit various IGF-mediated processes such as proliferation, survival, migration, and differentiation across different cell types. IGFBP-1, IGFBP-2, IGFBP-3, and IGFBP-5 may also improve IGF actions [38].

IGF-independent IGFBP functions have remained elusive but include their binding to various cell surface receptors, some of which have been identified, and activate intracellular signaling pathways [38]. Three IGFBPs—IGFBP-1, IGFBP-3, and IGFBP-5—are serine-phosphorylated in their central domains, while the others contain potential sites for serine/threonine kinase activity. Unphosphorylated IGFBP-1 can enhance IGF activity in specific cell types, but its phosphorylation increases its IGF-binding affinity, transforming it into an inhibitory form. The highly phosphorylated form in the bloodstream is the most abundant and likely plays a crucial role in rapidly regulating IGF bioavailability [51].

IGFBP-1 is expressed in multiple organs, with exceptionally high levels in the liver, where it plays a role in the acute metabolic regulation of IGF activity. IGFBP-1 is regulated in response to metabolic conditions, with insulin directly suppressing its gene transcription. As a result, fasting raises plasma IGFBP-1 levels, while feeding lowers them [52]. IGFBP-1 levels are elevated in individuals with T1D due to insulin deficiency. In contrast, in individuals with T2D, levels were disproportionately high relative to insulin due to hepatic insulin resistance. Furthermore, low fasting IGFBP-1 levels predicted the onset of prediabetes and T2D [53]. IGFBP-1 also facilitated the transdifferentiation of glucagon-producing *α*-cells into insulin-producing *β*-cells in the pancreatic islets, enhancing *β*-cell regeneration. This suggests a potential mechanism for IGFBP-1's protective role in preventing diabetes development [54].

IGFBP-2 is expressed predominantly in the liver, adipose tissue, and the reproductive and central nervous systems in adults, indicating its specialized functions in these organs. It has been found to influence metabolism and, more recently, adiposity [55]. IGFBP-2 levels were reduced in patients with T2D but elevated in those with T1D, likely reflecting the differences in insulin sensitivity between the two conditions [56]. In some animal studies, early-life hypermethylation of the IGFBP2 promoter and reduced IGFBP2 expression were linked to disrupted glucose regulation, obesity, and liver fat accumulation later in life [57].

IGFBP-3 binds IGF-I and IGF-II with equal affinity. IGFBP-3 has several IGF-independent actions, including inhibition of proliferation, survival, and migration and modulation of angiogenesis [58]. A recent study discovered that IGFBP-3 increased apoptosis in human hepatic sinusoid endothelial cells (HLSEC) and human umbilical vein endothelial cells (HUVEC) while decreasing the number of healthy cells, with more potent effects observed in HLSEC cells. This aligns with the development of HELLP syndrome, which is a severe variant of preeclampsia and is characterized by hemolysis, elevated liver enzymes, and low platelets [52]. Thus, an increased level of IGFBP-3 likely plays a role in HELLP syndrome by promoting apoptosis and impairing liver and endothelial function. Recent findings suggest that IGFBP-3 levels are significantly elevated in maternal serum in HELLP syndrome compared to normal pregnancies, supporting its potential role in the pathophysiology of this condition [59].

IGFBP-4 predominantly inhibits IGF actions in vitro. The protein acts as a cardiogenic growth factor and promotes stem cell differentiation into cardiomyocytes [60]. Almost half of the circulating IGFBP-5 is found in complexes with IGFs and ALS.

IGFBP-5 has a moderate binding affinity for IGF-II over IGF-I. It interacts with many cell surface receptors in human tissues, including testis, ovary, placenta, bone, lung, and uterus [61]. A specific cell surface receptor for IGFBP-5 has not been identified at the molecular level. However, functional interactions with specific cell surface proteins have been documented [62].

Unlike the other IGFBPs, IGFBP-6 has a 50-fold binding preference for IGF-II over IGF-I. Several studies indicated that IGFBP-6 expression is reduced in malignant cells compared to normal cells [63]. Beyond IGFBP-1 to IGFBP-6, other related proteins such as IGFBP-7and connective tissue growth factor (CTGF, also referred to as IGFBP-8) have been proposed as part of a broader IGFBP superfamily, though with lower binding affinity [64, 65].

## 4. IGF-Related Proteins in Human Pregnancy

During normal pregnancy, the maternal IGF system undergoes significant changes that play a crucial role in maternal adaptation and fetal development. In humans, maternal circulating IGF-I levels increase progressively throughout pregnancy. This rise supports maternal tissue growth and nutrient supply to the fetus [66]. Maternal IGF-II concentrations also rise during pregnancy, although the rise is less pronounced compared to IGF-I. IGF-II is more abundant than IGF-I in most species and is essential for placental development and function [66]. Maternal circulating IGFBP-1 concentrations are lower in early pregnancy but increase progressively as gestation progresses, eventually exceeding nonpregnant levels. This rise may influence IGF bioavailability and, consequently, fetal growth [67].

Higher concentrations of IGF-I improved placental development and nutrient transfer to the fetus. Conversely, IGF-II, mainly produced by the fetus, is crucial for fetal growth and development. Studies indicate that higher maternal IGF-I levels at mid- and late gestation are associated with greater placental and fetal growth, while IGF-II levels do not show the same correlation [68].

IGFBP-3 is the most abundant IGFBP in circulation and plays a key role in modulating IGF actions. During pregnancy, IGFBP-3 undergoes proteolytic cleavage, potentially reducing its capacity to bind IGFs and alter IGF bioavailability [69]. Although this process would theoretically increase IGF bioavailability, maternal serum levels of IGF-I and IGF-II actually increase, likely because of enhanced hepatic IGF production stimulated by placental hormones. This apparent paradox suggests the presence of compensatory mechanisms, which may include increased expression of other IGFBPs or stabilization via non-IGFBP mechanisms. Moreover, IGFBP fragments produced during proteolysis may retain partial IGF-binding capacity or modulate receptor interaction differently [66].

### 4.1. In Vivo and In Vitro Studies

The IGF/IGFBP system participates in various systemic and cellular functions in vivo and in vitro [70]. In humans, IGF-IR mutations lead to retarded intrauterine and postnatal growth [71]. IGF-regulated processes include wound healing, the development of the central nervous system and other tissues, and the regulation of protein, carbohydrate, and lipid metabolism. At the cellular level, IGF-I stimulates cell proliferation, survival, migration, and differentiation [72, 73]. For instance, IGF-I promotes myoblast differentiation into myocytes under specific conditions [74]. IGFs exhibit insulin-like effects and enhance insulin sensitivity. IGF-I can encourage glucose transport into cells [75], directly lowering blood glucose levels through IGF-IR [73]. It also amplifies insulin action by suppressing GH secretion from the pituitary, thus reducing its diabetogenic effects [76]. The role of the IGFBP system has been investigated in diabetic complications, including nephropathy. In diabetic conditions, alterations in the IGF/IGFBP system suggest a role in mediating IGF-I's biological activity [77, 78].

### 4.2. IGF-Related Proteins in the Human Placenta

The placenta is crucial in determining fetal growth, making it a significant factor in shaping lifelong health. IGF-I and IGF-II are found in nearly all placental cell types as early as 6 weeks of gestation and appear to play a role in almost every aspect of placental development and function [79].

IGFs are synthesized locally by placental cells and, in an autocrine/paracrine signaling manner, influence nearby cells to modulate placental and fetal development. IGF-II, a critical part of this system, regulates trophoblast invasion and vascular remodeling, processes that are essential for establishing and maintaining adequate placental perfusion to ultimately deliver oxygen and nutrients to the fetus [80]. Diabetes can impair IGF-II-driven paracrine signaling, contributing to complications like preeclampsia or placental insufficiency [81]. Paracrine IGF-I signaling enhances glucose uptake and amino acid transport in the placenta, ensuring the maternal nutrient supply meets fetal growth demands [82]. Autocrine IGF signaling also contributes to trophoblast survival and proliferation in the placenta, supporting placental development and function [83].

Fetal tissues mainly express IGF-II mRNA, whereas maternal tissues (decidua) produce IGFBP mRNAs, with ligand–receptor interactions believed to regulate critical processes [82]. Decidual IGFBP-1 can become phosphorylated and may then regulate trophoblast migration and invasion. It was also suggested to modulate placental development and regulate placental vascularization and nutrient transport. Alterations in IGFBP-1 phosphorylation have been linked to diabetes pregnancies and adverse fetal outcomes [84]. Both IGFs enhance the proliferation and survival of placental fibroblasts. Moreover, IGF-I can prevent fibroblast apoptosis [85]. In addition, IGF-I regulates the differentiation of cytotrophoblasts into the syncytiotrophoblast [86]. In first-trimester placentas, IGF-II mRNA is expressed in the cytotrophoblast but is absent in the syncytiotrophoblast. Extravillous trophoblast cells express abundant IGF2R [87]. A key difference in the chorionic villous between the second and third trimesters is that villous cytotrophoblasts no longer express IGF-II mRNA, indicating that IGF-II may influence cytotrophoblast growth and differentiation in a time-specific manner [82]. In the second and third trimesters, decidual cells in the basal plate region express mRNA for all seven IGFBPs, with IGFBP-1 being the most highly expressed [88].

IGF-IR is found in all cell types, including the trophoblast, villous endothelium, and mesenchymal core [89], while IGF2R is expressed in the extravillous cytotrophoblast and the syncytiotrophoblast [90]. IR expression changes in a spatiotemporal manner during pregnancy. In the first trimester, IRs are primarily located on the microvillous membrane of the syncytiotrophoblast facing maternal circulation, suggesting yet undefined roles of maternal insulin on the placenta. Some are also found in villous cytotrophoblasts. At term of pregnancy, IRs are mainly expressed on the placental endothelium, facing the fetal circulation. This differs from the IGF-IR, which does not show this spatiotemporal shift in its location [91]. Changes in IGF-IR signaling act as a switch for lineage commitment while preserving cell proliferation, ensuring the balance between self-renewal and differentiation [48].

Placental hormones, human placental lactogen (hPL), and placental growth hormone (PGH) play significant roles in regulating maternal metabolism and fetal development through their interactions with IGFs. The positive correlation between PGH and IGF-I concentrations in maternal blood suggests a regulatory role of placental growth factors for IGF-I production [92]. Indeed, during gestation, PGH becomes the dominant regulator of maternal IGF-I production, as pituitary GH secretion is suppressed [93]. This transition increases IGF-I levels in maternal tissues, promoting metabolic adaptations that support fetal growth. PGH and hPL work together to enhance IGF production and regulate intermediary metabolism, increasing the supply of glucose and amino acids to the fetus. This collaboration ensures that the developing fetus receives sufficient nutrients for proper growth and development [94]. PGH and hPL induce adaptive changes such as increased insulin resistance, expanded pancreatic beta-cell mass, and greater insulin secretion [95]. These adjustments help maintain maternal euglycemia while ensuring a consistent placental glucose supply [96]. Disruptions in these mechanisms are associated with altered IGF signaling, potentially resulting in fetal growth abnormalities, including macrosomia or intrauterine growth restriction [80].

The significance of total IGF levels during pregnancy is well established; the bioactive fraction of IGF—free IGFs is crucial for mediating physiological effects. In pregnancies with diabetes, hyperglycemia and insulin resistance can disturb the equilibrium between free and bound IGFs. Increased maternal insulin levels in GDM have been associated with changes in IGFBP production, leading to altered IGF bioavailability [97]. IGFBP-1 levels inversely correlate with maternal insulin levels and can influence fetal nutrient availability [98]. The activity of binding proteins depends on proteases. Pregnancy-associated plasma protein-A (PAPP-A) is a protease that plays a critical role in cleaving IGFBPs, especially IGFBP-4. This cleavage releases free IGFs, allowing them to interact with IGF receptors. In diabetic pregnancies, abnormal PAPP-A activity has been implicated in altered IGF signaling.

IGFs can be identified in various fetal tissues starting from the first trimester [99]. Circulating levels of fetal IGF-I and IGF-II both increase with gestational age, with IGF-II concentrations consistently being higher than those of IGF-I [100]. At the term of pregnancy, IGF-I levels are directly correlated with birth weight [101]. In samples obtained from cordocentesis, fetal IGFBP-1 levels remained virtually unchanged in the second and third trimesters [102].

## 5. Placental IGF-Related Proteins in Patients With Diabetes

### 5.1. Prediabetes

Prediabetes has preceded the onset of overt diabetes for many years. Adults with prediabetes often may be asymptomatic, while laboratory tests already indicate metabolic abnormalities such as elevated blood glucose levels without meeting the criteria for diabetes. The diagnostic criteria for prediabetes are not uniform in different scientific societies. Usually, prediabetes is defined by a fasting glucose level of 5.6 to 6.0 mmol/L (100–125 mg/dL) and a glucose level of 7.7 to 11 mmol/L (140–199 mg/dL) measured 2 h after a 75-g oral glucose load, or glycated hemoglobin A1C level (HbA1C) of 5.7% (39 mmol/mol) to 6.4% (46 mmol/mol) or 6.0%–6.4% (42–46 mmol/mol) [103]. It is estimated that 5%–10% of people progress to overt diabetes each year, depending on region and ethnicity [104]. A meta-analysis found that prediabetes was associated with increased mortality and increased cardiovascular event rates (excess absolute risk, 7.36 per 10,000 person-years for mortality and 8.75 per 10,000 person-years for cardiovascular disease during 6.6 years) [5].

Prediabetes during pregnancy has become an increasingly recognized condition that poses a significant risk to both maternal and fetal health. The prevalence of prediabetes in pregnant women is concerning, especially given its association with GDM and other adverse pregnancy outcomes. Research indicates that women with prediabetes are at heightened risk for developing GDM, which can lead to complications such as hypertensive disorders and increased cesarean delivery rates [105, 106]. Decreased insulin sensitivity has been associated with elevated levels of IGFBPs, prompting the hypothesis that changes in IGFBP expression may be biomarkers of maternal insulin resistance during pregnancy [107]. Hyperglycemic conditions, such as those associated with prediabetes, can lead to increased production of metabolites within the placenta. This is often connected with an upregulation of proinflammatory cytokines, which can contribute to placental dysfunction and apoptosis, as shown in animal studies [108].

### 5.2. PGDM

PGDM refers to diabetes present before the onset of pregnancy, encompassing both T1D and T2D. It is a condition characterized by elevated blood glucose levels that can lead to various complications for both mother and fetus. The increasing global prevalence of PGDM raises concerns regarding its impact on pregnancy outcomes and fetal development. The association between PGDM and adverse pregnancy outcomes is well documented, with studies indicating that women with PGDM face heightened risks of congenital anomalies, particularly cardiac defects, macrosomia, and preterm birth [109, 110]. The pathophysiology underlying the adverse effects observed in PGDM on fetal development is multifactorial. It is known that hyperglycemia in critical periods of organogenesis can impair normal cellular processes, leading to developmental or even teratogenic defects [56, 111]. These may be due to oxidative stress, inflammation, and altered growth factor signaling pathways, including those mediated by IGFs [112].

Placental function is altered in diabetes [113]. In PGDM, placental expression of various genes, including those related to glucose transport and angiogenesis, may be dysregulated. In contrast to prediabetes and GDM, PGDM pregnancies are characterized by a more severe impairment of glucose homeostasis caused by frequent glucose fluctuations despite intensive insulin therapy [114]. This results in elevated glucose transfer through the placenta. Transient hyperglycemic episodes cause an increase in GLUT-1 transporter expression in the placenta [115]. GLUT-1 expression in trophoblasts remains relatively stable under moderate glucose concentrations, but transient hyperglycemic episodes can induce increased transporter expression in PGDM, thereby enhancing the placenta's glucose uptake [116]. This increased uptake may reflect increased placental glucose demand to cover energy requirements for biosynthetic activities, which may be related to the elevation of key growth regulators (e.g., IGF-I and IGF-II) in the placenta, which themselves may contribute to upregulation of GLUT-1 expression [28, 114, 116].

In newborns of women with T1D, higher levels of IGFBP-3 and IGF-II in the umbilical cord are often associated with macrosomia [117, 118]. In pregnancies complicated by T1D, the cord serum concentrations of highly phosphorylated IGFBP-1 were lower than in the controls [98]. In pregnancies with T1D, highly phosphorylated IGFBP-1 concentrations were lower in pregnant women with T1D or GDM than in the control group of women with standard glucose tolerance [119]. A similar situation is observed in T2D. Studies indicated that in large for gestational age (LGA) babies born to mothers with T2D, neonatal IGFBP-1 concentrations in cord blood were significantly reduced [116]. In the third trimester, circulating IGF-1 levels were lower in mothers with T1D, and maternal and fetal IGFBP-3 concentrations were higher [117]. Levels of IGF-I and IGFBP-3 in the cord blood of newborns from mothers with T1D and T2D were higher than those from mothers without diabetes. In this study, cord blood IGF-I and IGF-II levels were associated, and both correlated with maternal HbA1C levels. However, cord IGFBP-1 concentrations were notably elevated in pregnancies with T1D and T2D [28]. The interplay between maternal metabolic status, IGF signaling, and fetal development underscores the importance of maintaining glycemic control before and during pregnancy to mitigate risks associated with PGDM [120].

### 5.3. GDM

GDM is defined as a state of glucose intolerance and hyperglycemia first diagnosed during pregnancy [121, 122]. It belongs to the most frequent complications of pregnancy, affecting approximately 15% of pregnant women [123]. The most common risk factors for GDM include advanced maternal age, overweight and obesity, lack of physical activity, family history of T2D, history of GDM, previous delivery of a macrosomic newborn, arterial hypertension, and polycystic ovary syndrome. While the pathogenesis of GDM remains unclear, it is evident that beta-cell dysfunction and the failure of insulin secretion to compensate for insulin resistance induced by pregnancy are significant contributing factors [124]. GDM may include undiagnosed pre-existing T2D. Therefore, the increasing prevalence of GDM is likely to reflect the underlying epidemic of T2D in the population. Conversely, women with a history of GDM have a sevenfold increased risk of developing T2D in later life. Typically, GDM is diagnosed between 24 and 28 weeks of gestation, although metabolism may already be altered before 20 weeks of gestation in pregnancies subsequently diagnosed with GDM.

Because of their crucial role in regulating glucose metabolism and insulin sensitivity [125], the IGF/IGFBP system may strongly contribute to the pathogenesis of GDM and related complications. Clinical studies of GDM pregnancies found an increase in maternal IGF-I levels, a decrease in cord blood IGF-I levels, and a positive correlation between maternal insulin and fetal IGF-I concentrations and the birth weight of a newborn [126]. Maternal IGF-I and IGF-II levels show distinct dynamic changes throughout pregnancy. While both pregnant women with and without GDM exhibited an increase in serum IGF-I levels during pregnancy, followed by a significant decline after delivery, the elevation of serum IGF-II levels persisted in pregnant women with GDM for a 6–12-month postpartum period, exhibiting a notable elevation compared to the non-GDM group [126]. Pregnant women had higher levels of IGF-I and IGF-II in their systemic circulation than nonpregnant women, and there was no significant difference between women with GDM and healthy pregnant controls [127]. However, another study found elevated serum levels of IGF-I in women with GDM throughout gestation compared to healthy pregnant controls [128]. These conflicting findings may reflect methodological and design differences across studies, including different antibodies for quantification of IGFs and IGFBPs, as well as different study population characteristics.

It was proposed that the changes in the IGF axis may be implicated in the pathogenesis of GDM, with significant associations and incremental predictive value detected as early as Gestational Weeks 10–14, which is about 14 weeks earlier than GDM is typically diagnosed [128].

Maternal IGFBP-1 levels are slightly, but significantly, decreased in GDM [129], with the decrease found already during the second trimester before GDM diagnosis [130]. At 13 weeks of gestation, maternal concentrations of free, that is, not bound to IGFBPs, IGF-I and IGFBP-1 were inversely associated with GDM diagnosis later on [131]. Cord blood IGFBP-1 levels were inversely related to birth weight. Also, maternal IGFBP-2 is inversely associated with GDM in every trimester of pregnancy. Concentrations of IGFBP-3, the primary IGFBP in serum that serves as a circulating reservoir for IGF-I, were increased in pregnant and nonpregnant women [132]. Its serum levels declined throughout pregnancy and following delivery. IGFBP-3 serum levels were significantly elevated in women with GDM compared to healthy pregnant controls. The data from a single study indicated that maternal IGFBP-3 levels in late gestation were higher in women with GDM than in those without. However, this association was not observed in early or midgestation. Other IGF/IGFBP system components have demonstrated no clear or consistent associations with GDM. The alterations in circulating IGF levels during mid and late gestation may either contribute to the cause or be a consequence of GDM and may have the potential to be used as a biomarker for later GDM diagnosis [130].

## 6. Associations With Gestational Outcomes

Macrosomia, or LGA, is a prevalent complication in pregnancies affected by diabetes. Also, fetal growth restriction (FGR) and small for gestational age (SGA) neonates tend to be more common in pregnancies with diabetes because of underlying maternal vascular disease [112]. Given the association between macrosomia and an increased risk of adverse outcomes for both mother and offspring, accurate antenatal prediction of fetal macrosomia or LGA could prove beneficial in guiding appropriate models of care and interventions that may prevent or reduce these associated risks. Nevertheless, current prediction strategies, including physical examination and ultrasound assessment of fetal growth, are not precise enough. Fetal hyperglycemia and hyperinsulinemia are distinctive characteristics of such fetuses in utero and have long been regarded as significant drivers of fetal overgrowth [128]. IGF-I concentrations in cord blood are higher in neonates born to mothers with both GDM and PGDM than those born to mothers with adequate glucose tolerance. A meta-analysis of 11 observational studies demonstrated that cord blood IGF-I levels were markedly elevated in LGA compared to AGA (appropriate for gestational age) neonates [120, 129]. These data were taken as evidence to suggest a contribution of IGF-I to fetal macrosomia. However, IGF-I is generally regarded as the predominant growth stimulator in postnatal life, while IGF-II facilitates growth during the fetal stage [130]. The potential role of IGF-II in the development of fetal macrosomia has remained unclear. Some studies have indicated that IGF-II concentration in cord blood is elevated in infants born to mothers with diabetes [29, 130, 132], but this may reflect bystander rather than causal effects. A study in pregnant women with T1D failed to find differences in maternal IGF-I levels throughout gestation between macrosomic neonates and neonates with appropriate birth weights [130]. Recent studies have further explored the potential role of IGF-related proteins for adverse pregnancy outcomes. In GDM, IGF bioavailability is altered emphasizing the role of IGFBPs in modulating IGF function and fetal growth [133]. This makes them potentially useful biomarkers also for predicting fetal macrosomia in both diabetic and nondiabetic pregnancies. In particular, maternal IGFs, IGFBP-1 and IGFBP-3, potentially have clinical relevance [134] and should be tested further in future studies.

Maternal plasma triglyceride concentrations in the second trimester and cord blood IGF-I concentrations were elevated in pregnant women with LGA neonates despite the absence of significant differences between AGA and LGA neonates in cord blood IGF-II, IGFBP-1, and IGFBP-3 levels [29]. The adjusted models showed a significant association between maternal plasma triglyceride concentrations and birth weight *z*-scores, which are related to cord blood IGF-I concentrations. Maternal free palmitic acid and stearic acid concentrations, but not oleic or linoleic acid, were significantly associated with cord blood IGF-I concentrations [135].

Amniotic fluid (AF) is another fetal compartment in which the IGF system has been studied. AF concentrations of IGFBP-1 increased gradually between 14 and 20 weeks and correlated with AF IGF-I and IGF-II levels [136]. High second-trimester AF concentrations of IGFBP-1 were associated with lower birth weight. However, high IGFBP-1 levels did not predict SGA neonates but were associated with lower placenta weight [137]. Recently, lower AF IGFBP-3 concentrations were associated with FGR [138]. AF concentrations of IGFBP-1, the binding protein in the highest concentration in AF [139, 140], were negatively associated with birth weight [141] and are lower in LGA than in AGA infants and in infants with macrosomia compared to those with low birth weight. AF IGFBP-3 concentrations were positively related to birth weight, suggesting that both binding proteins may emerge as important early prognostic factors of fetal growth factors [139]. However, another study failed to confirm the utility of IGFBP-1 concentrations as birth weight predictors [142].

While Table 1 focuses on canonical IGF-related proteins, it is important to note that IGFBPs can also form complexes with other circulating plasma proteins, modulating IGF transport, stability, and receptor accessibility. Weinzimer et al. [149] demonstrated that transferrin specifically binds to IGFBP-3, forming stable complexes that may influence the distribution and function of IGFBP-3 in the circulation. Similarly, Šunderić et al. [140] showed that IGFBP-2 interacts with *α*2-macroglobulin, one of the major protease inhibitors in human plasma, suggesting that this association may prolong IGFBP-2 stability and alter its IGF-modulating activity. In addition, Westwood et al. [150] identified *α*2-macroglobulin as a novel component of the IGF/IGFBP-1 axis, reporting that IGFBP-1 can form complexes with *α*2-macroglobulin, potentially regulating IGF bioavailability in maternal–fetal circulation. Further, Gligorijević and Nedić [151] provided evidence that fibrinogen interacts with IGFBP-1 under physiological plasma conditions, raising the possibility that coagulation-related proteins may also contribute to IGF system regulation. Taken together, these studies broaden our understanding of the IGFBP molecular interactions and highlight that IGFBPs may regulate IGF actions not only through classical ligand binding but also via associations with other plasma proteins, which could be particularly relevant in pregnancy and diabetes.

In fetal overgrowth and pregnancies with SGA neonates, the placental IGF/IGFBP axis is altered [101]. Placental IGF-I mRNA is decreased, and expression levels of all IGFBPs increased in SGA compared to AGA neonates. These changes were associated with alterations in DNA methylation levels of IGF-I (hypermethylated) and IGFBPs (hypomethylated). Thus, epigenetic modifications may be pivotal regulators of the placental IGF/IGFBP axis and may affect placental or fetal growth in SGA neonates. This differs from pregnancies with LGA neonates, in which corresponding changes in the respective gene promoters' methylation could not explain gene expression changes [101].

The relationship between maternal IGF-I and fetal growth across the different types of diabetes is a crucial area that requires further investigation and clarification. A comparative study of women with T1D, T2D, GDM, and controls found that the median maternal IGF-I values in the third trimester were not significantly different between the groups [139]. This opposes other studies discussed above and underscores the need for more research in this area (Table 1).

## 7. Conclusions

This review highlights the multifaceted role of IGFs and IGFBPs during pregnancy, particularly in those pregnancies complicated by diabetes. IGFs, notably IGF-I, IGF-II, and IGFBPs, are critical in fetal and placental growth.

These effects underscore the complex relationship between maternal metabolism, placental function, and fetal development in diabetes, with implications for birth weight and neonatal health. Moreover, the findings suggest that the impact of IGFs extends beyond pregnancy and may influence disease risk in later life.

In addition, we included the state of prediabetes and the impact of hyperglycemia on the placenta, characterized by increased production of metabolic byproducts, which may impair placental growth and nutrient transport. Placental changes may disrupt the IGF/IGFBP axis, which is essential for proper placenta and fetus development. They may lead to altered expression of IGF receptors and binding proteins. Understanding these pathways might contribute to developing preventive interventions to address maternal and fetal health, though the outcomes and feasibility remain uncertain. Further research is needed to evaluate whether the modulation of IGF/IGFBP signaling pathways can serve as viable therapeutic targets for reducing adverse outcomes in diabetic pregnancies. These studies should explore the structural modifications of IGF-related proteins, such as glycation and glycosylation, particularly under hyperglycemic conditions. These posttranslational modifications may significantly alter IGF signaling and could serve as early biomarkers of placental dysfunction or fetal overgrowth in diabetic pregnancies.

## Figures and Tables

**Table 1:** Associations of IGF-related proteins with gestational outcomes.

**First author, year [reference]**	**Analyzed parameter**	**Clinical characteristics** **Number of study participants**	**Main findings**
Chevallier, 1998 [143]	IGFBP-1	58 AF samples from 13 to 19 gestational weeks	Negative correlation of IGFBP-1 in the second trimester with birth weight; positive relationship between IGFBP-1 at term with birth weight

Hakala-Ala-Pietila, 1993 [144]	IGFBP-1	AF samples (148) and maternal serum samples (129) from 15 to 16 gestational weeks	Elevated AF IGFBP-1 levels were related to FGR; levels of IGFBP-1 in maternal serum were not related to FGR

Verhaeghe, 1999 [142]	IGF-I, IGF-II, IGFBP-1, and C-peptide in second trimester AF	209 AF samples with normal fetal karyotype between 14 and 20 weeks	No correlation of AF levels with birth weight; birth weight percentile distribution was comparable in two groups (SGA and LGA) of newborns with AF values of IGF-I, IGF-II, IGFBP1, or C-peptide

Nawathe, 2016 [101]	(1) Placental mRNA and protein of IGF-I, IGF-II, IGFBP-1, IGFBP-2, IGFBP-3, IGFBP-4, and IGFBP-7 (2) CpG methylation of IGF-I promoter regions	(1) 74 women, AGA (*n* = 38), SGA (*n* = 16), and LGA (*n* = 20) neonates (2) 24 placentas (8 in each group)	(1) Placental mRNA and protein levels of IGF1 were lower and of IGFBPs were higher in SGA compared to AGA; in LGAs, placental mRNA and protein levels of IGF1 were not different and those of IGFBPs were lower compared to AGA (2) IGF-I methylation higher in SGA than AGA neonates

McIntyre, 2000 [145]	GHBP, PGH, IGF-I, IGF-II, IGFBP-1, IGFBP-2, and IGFBP-3 measured in maternal serum at 28–30 gestation week or 36–38 gestation week	Third-trimester samples from pregnancies complicated with T1D (*n* = 13), T2D (*n* = 6), and FGR (*n* = 16)	In third-trimester maternal IGF-1 measurements in women with PGDM, IGF-I was significantly positively associated with macrosomia

Grissa, 2010 [146]	Maternal blood after delivery (before placenta delivery) and cord blood samples; IGF-I, IGFBP-3, FGF-2, EGF, and PDGF were quantified	Women with GDM (*n* = 30) and their macrosomic neonates (*n* = 30), healthy pregnant women (*n* = 30) and their neonates (*n* = 30)	IGF-I, IGFBP-3, EGF, FGF-2, and PDGF concentrations were higher in maternal serum and cord blood serum of GDM women and their macrosomic babies than in their respective controls

Simmons, 1995 [147]	Umbilical cord blood–insulin, C peptide, fructosamine, SHBG, sex hormone, IGF-I; neonatal anthropometry measured within 24 h after delivery	Neonates from normal (*n* = 125) pregnancies and from those with GDM (*n* = 35)	Serum IGF-I levels significantly higher in GDM women and their macrosomic neonates than in mothers without GDM and neonates from these pregnancies

Tisi, 2004 [148]	IGF-II, IGFBP-1, and IGFBP-3 in AF at 12-20 weeks	543 mother–infant dyads	AF IGFBP-3 was positively associated with birth weight within LGA and macrosomia compared with AGA infants

Abbreviations: AF, amniotic fluid; AGA, appropriate for gestational age; CpG, cytosine–phosphate–guanine; EGF, epidermal growth factor; FGF, fibroblast growth factor; FGR, fetal growth restriction; GHBP, growth hormone binding protein; LGA, large for gestational age; PDGF, platelet-derived growth hormone; PGH, placenta growth hormone; SGA, small for gestational age; SHBG, sex hormone binding globulin.
